# Antiosteoporotic Activity of Anthraquinones from *Morinda officinalis* on Osteoblasts and Osteoclasts

**DOI:** 10.3390/molecules14010573

**Published:** 2009-01-23

**Authors:** Yan-Bin Wu, Cheng-Jian Zheng, Lu-Ping Qin, Lian-Na Sun, Ting Han, Lei Jiao, Qiao-Yan Zhang, Jin-Zhong Wu

**Affiliations:** 1 Department of Pharmacy, Fujian University of Traditional Chinese Medicine, Fuzhou, Fujian 350108, P.R. China; E-mail: wxsq1@163.com (Y-B. W.); 2 Department of Pharmacognosy, School of Pharmacy, Second Military Medical University, Shanghai 200433, P.R. China; 3 Academy of Integrative Medicine, Fujian University of Traditional Chinese Medicine, Fuzhou, Fujian 350108, P.R. China

**Keywords:** *Morinda officinalis*, Anthraquinones, Osteoblast, Osteoclast, Osteoporosis.

## Abstract

Bioactivity-guided fractionation led to the successful isolation of antiosteoporotic components, i.e. physicion (**1**), rubiadin-1-methyl ether (**2**), 2-hydroxy-1-methoxy- anthraquinone (**3**), 1,2-dihydroxy-3-methylanthraquinone (**4**), 1,3,8-trihydroxy-2-methoxy- anthraquinone (**5**), 2-hydroxymethyl-3-hydroxyanthraquinone (**6**), 2-methoxyanthraquinone (**7**) and scopoletin (**8**) from an ethanolic extract of the roots of *Morinda officinalis*. Compounds **4-8** are isolated for the first time from *M. officinalis.* Among them, compounds **2** and **3** promoted osteoblast proliferation, while compounds **4, 5** increased osteoblast ALP activity. All of the isolated compounds inhibited osteoclast TRAP activity and bone resorption, and the inhibitory effects on osteoclastic bone resorption of compounds **1** and **5** were stronger than that of other compounds. Taken together, antiosteoporotic activity of *M. officinalis* and its anthraquinones suggest therapeutic potential against osteoporosis.

## Introduction

*Morinda officinalis* How (Rubiaceae) is a small vine that grows widely in tropical and subtropical regions. The roots of this plant (named Bajitian) have been recorded in pharmacopeia of the People’s Republic of China and used to help strengthen the bones and kidneys and enhance the immune system function. This plant has also been used in traditional medicine in northeast Asia to treat impotence, menstrual disorders, and inflammatory diseases such as rheumatoid arthritis and dermatitis for over 2,000 years [1]. Seo reported that *Morinda* root extracts may act as both a suppressor of bone resorption and an enhancer of bone formation *in vivo*, and may have some favorable effects for preventing and treating the osteoporosis induced by sciatic neurectomy [2]. During the course of our search for natural products capable of preventing menopausal osteoporosis, we have investigated how ethanolic extracts of *M. officinalis* roots prevent and treat bone loss of ovariectomized rats. These bioactivities prompted us to continue to investigate its antiosteoporotic chemical components. 

In osteoporosis, the formation and function of osteoblasts decreases whilst osteoclast formation and recruitment increases, and this causes a relative increase of osteoclastic bone resorption over osteoblastic bone formation. The bone formation is related to osteoblastic proliferation, alkaline phosphatase (ALP) activity, osteocalcin and collage synthesis; and the bone resorption is associated with osteoclast formation and differentiation, and tartrate-resistant acid phosphatase activity (TRAP). In this study, we used osteoblast and osteoclast induced from bone marrow cells to screen antiosteoporotic chemical constituents, and aims to search compounds for improving bone formation or inhibiting bone resorption from *M. officinalis*.

## Results and Discussion

The ethanolic extract of the roots of *M. officinalis* was further fractionated into petroleum ether, ethyl acetate, *n*-BuOH and H_2_O fractions. Among them, the ethyl acetate fraction dose dependently stimulated osteoblast proliferation and ALP activity, and inhibited osteoclastic TRAP activity. At a concentration of 20 µg/mL, this active fraction increased osteoblast proliferation and ALP activity respectively by 90.1% and 33.9%, and inhibited osteoclast TRAP activity by 55.0% (Table 1). 

**Table 1:** Effects of various fractions of ethanolic extracts of *Morinda officinalis* (EMO) on osteoblast and osteoclast (n=8, x±SD).

Fractions	Conc. (µg/mL)	Osteoblast proliferation Absorbance (550nm)	Osteoblastic ALP activity (μmol/mg)	Osteoclastic TRAP activity (nmol/min/100 Osteoclastic cell )
Control		0.51±0.03	3.71±0.31	15.0±1.3
EMO extracts	200	0.76±0.05^**^ (49.1%)	4.37±0.42^**^ (17.8%)	9.2±0.9^**^ (38.7%)
	400	0.86±0.08^**^ (68.6%)	4.42±0.39^**^ (19.1%)	8.8±0.6^**^ (41.3%)
Petroleum ether fraction	10	0.54±0.04 (5.8%)	3.31±0.29 (49.1%)	14.0±1.4 (-6.7%)
	20	0.51±0.02 (0%)	3.38±0.32 (-1.6%)	13.2±1.1 (-12.0%)
Ethyl acetate fraction	10	0.79±0.06^**^ (54.9%)	4.40±0.43^**^ (18.6%)	8.0±0.7^**^ (90.1%)
	20	0.97±0.11^**^ (90.1%)	4.97±0.48^**^ (33.9%)	6.8±0.8^**^ (55.0%)
*n*-Butanol fraction	10	0.61±0.04 (19.6%)	3.38±0.29 (-8.9%)	13.8±0.6 (-8.0%)
	20	0.58±0.01 (13.7%)	3.39±0.32 (-8.6%)	15.2±1.6 (1.3%)
Aqueous fraction	10	0.52±0.02 (1.9%)	3.30±0.31 (-11.1%)	16.1±1.3 (7.3%)
	20	0.56±0.06 (9.8%)	3.41±0.33 (-8.1%)	15.5±1.4 (3.3%)

The data in brackets shows the percentage of increase or decrease compared with control.

**Figure 1:**
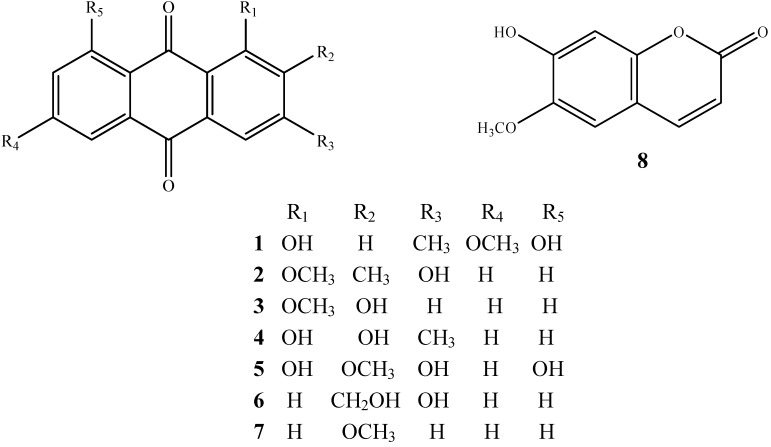
Structures of compounds **1-8** isolated from root of *Morinda officinalis.*

Based on the above results, activity-guided fractionation of the ethyl acetate fractions was carried out for the isolation of active constituents. Further fractionation and separation by several chromatographic methods yielded seven anthraquinones and one coumarin. The structures of these compounds were identified as physcion (**1**) [3], rubiadin-1-methyl ether (**2**) [1], 2-hydroxy-1-methoxy- anthraquinone (**3**) [1], 1,2-dihydroxy-3-methylanthraquinone (**4**) [4], 1,3,8-trihydroxy-2-methoxy- anthraquinone (**5**) [5], 2-hydroxymethyl-3-hydroxyanthraquinone (**6**) [6], 2-methoxyanthraquinone (**7**) [7] and scopoletin (**8**) [3], respectively, by comparison of their spectroscopic data (^1^H-, ^13^C-NMR and MS) with those reported in the literature. Compounds **4-8** are isolated and identified for the first time from the *M. officinalis*. Their structures are shown in Figure 1.

Osteoblastic bone formation is thought to be mediated by two different processes: one is the formation of new osteoblasts, and the other is the activity of osteoblasts to produce bone matrix. Osteoblasts produce collagen, alkaline phosphatase, osteocalcin and other matrix proteins to form new bone [8]. Seven anthraquinones and one coumarin were isolated from the biologically active ethyl acetate soluble phase. These compounds were tested for stimulatory effects on osteoblasts at concentrations of 10^-10^, 10^-9^ and 10^-8^ mol/L. Compound **3** stimulated osteoblastic proliferations, but did not show any activity on osteoblast alkaline phosphatase (ALP), while compounds **4** and **5** did not stimulate osteoblastic proliferation, but significantly increased the alkaline phosphatase activity of osteoblasts. The coumarin scopoletin produced stimulatory effects both in osteoblast proliferation and alkaline phosphatase activity. Compounds **1, 2, 6** and **7** did not show any activity on osteoblasts at the same concentrations (Table 2). 

**Table 2:** Effects of compound **1-8** on osteoblast proliferation and ALP activity in neonatal rat calvaria cultures (n=8, x±SD).

Compound	Osteoblast proliferation absorbanc (550 nm)			Osteoblastic ALP activity (μmol/mg)		
	10^-10^mol/L	10^-9^mol/L	10^-8^mol/L	10^-10^mol/L	10^-9^mol/L	10^-8^mol/L
**Control**	0.55±0.02	0.55±0.02	0.55±0.02	3.91±0.37	3.91±0.37	3.91±0.37
**1**	0.52±0.01	0.57±0.04	0.54±0.03	3.54±0.35	3.60±0.34	3.53±0.29
**2**	0.54±0.08	0.53±0.06	0.55±0.05	3.53±0.31	3.60±0.34	3.71±0.36
**3**	0.62±0.04^*^	0.63±0.05^*^	0.65±0.04^*^	3.65±0.28	3.60±0.26	3.16±0.36
**4**	0.52±0.02	0.55±0.02	0.62±0.02^*^	4.49±0.36^**^	4.60±0.43^**^	4.75±0.39^**^
**5**	0.64±0.09	0.59±0.06	0.56±0.02	3.47±0.46	4.99±0.47^**^	5.20±0.46^**^
**6**	0.55±0.03	0.5±0.05	0.60±0.02	3.98±0.30	3.80±0.34	3.56±0.34
**7**	0.43±0.02	0.51±0.04	0.54±0.03	3.67±0.26	3.54±0.31	3.81±0.36
**8**	0.66±0.05^*^	0.67±0.03^*^	0.69±0.03^**^	4.79±0.46^**^	4.78±0.37^**^	5.09±0.47^**^

*P < 0.05, **P < 0.01, compared with control

**Table 3:** Effects of compound **1-8** on osteoclast TRAP activity induced from rat marrow cells (n=8, x±SD).

Compound	Osteoclastic TRAP activity (nmol/min/100 osteoclastic cell )		
	10^-7^mol/L	10^-6^mol/L	10^-5^mol/L
**Control**	34.3±2.8	34.3±2.8	34.3±2.8
**1**	30.7±2.9^*^	19.9±1.5^**^	17.7±1.4^**^
**2**	21.6±1.8^**^	18.5±1.4^***^	13.8±1.3^***^
**3**	33.8±1.4	28.6±2.8^*^	20.7±2.4^**^
**4**	27.4±1.9^**^	26.6±1.3^**^	25.0±1.4^**^
**5**	22.4±2.2^**^	17.8±1.7^***^	9.8±1.0^***^
**6**	28.9±2.1^*^	26.80±2.0^**^	20.3±1.4^**^
**7**	16.5±1.6^***^	16.0±1.5^***^	13.5±1.1^***^
**8**	28.4±2.1^*^	25.1±1.9^**^	17.6±1.7^***^

*P < 0.05, **P < 0.01, ***P < 0.001 compared with control

**Figure 2:**
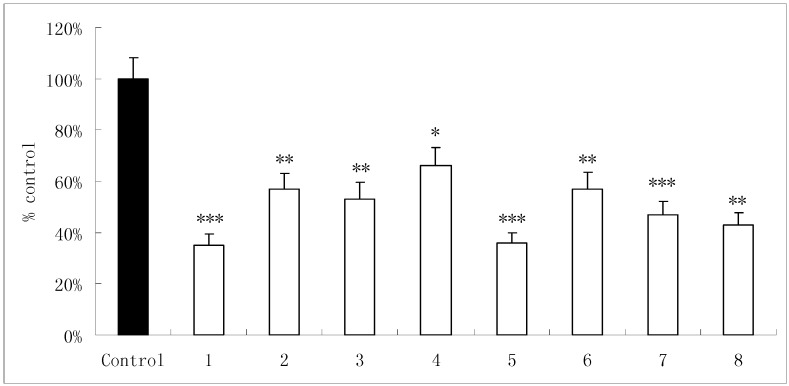
The inhibitory effects of compound **1-8** on the resorption pit area on bone slices formed by osteoclasts in cultures.

Osteoclastic bone resorption is mediated by the formation of new osteoclasts and the resorption activity of osteoclasts. The activity of resistant tartaric acid phosphatase is directly related with osteoclastic bone resorption [9]. In the process of co-culture of osteoclasts and bone slices, osteoclasts lyse the inorganic salt and organic substances of bone slices to form resorption pits on bone slices. Compounds **1-8** dose-dependently inhibited osteoclastic TRAP activity, and reduced osteoclastic bone resorption pit area on bone slices. After treatment of osteoclasts for 10 days with compounds **1-8** at a concentration of 10^-6^ mol/L, the osteoclastic bone resorption pit area on bone slices was reduced by 65%, 43%, 47%, 34%, 64%, 43%, 53%, and 57%, respectively, as compared to the control (Table 3; Figure 2).

*P < 0.05, **P < 0.01, ***P < 0.001 compared with control

Consistent with our study, some anthraquinones have been reported to have antiosteoporotic activity. Emodin, a naturally occurring anthraquinone present in the roots and bark of numerous plants of the genus *Rhamnus* could increase bone formation through activating the mRNA expression of bone morphogenetic protein (BMP)-2 and alkaline phosphatase (an early marker of osteoblast differentiation) and the mineralization in the differentiation process of mouse osteoblastic MC3T3-E1 [10]. Diacerein has been shown to inhibit osteoclastic bone destruction through the inhibition of RANKL expression and the increase of OPG expression in MC3T32 E1 cells [11]. Two newly synthesized anthraquinone molecules have been shown to increase the trabecular bone volum and trabecular thickness in an *in vivo* study [12]. These results together with our present study suggest the therapeutic potentials of anthraquinones in osteoporosis, and provide a lead in the early drug discovery and development for osteoporosis.

All seven anthraquinones isolated from *Morinda officinalis* have the same anthraquinone skeleton, and only differ in the substitutions at the C-1, C-2, C-3, C-6 and C-8 positions. The substituent may be a hydroxyl, methyl, or methoxyl group. Compound **3**, which has a methoxyl group at C-1 and a hydroxyl group at C-2, stimulated osteoblast proliferation, and did not increase osteoblast ALP activity. Compounds **4** and **5**, which have hydroxyl groups at C-1, and hydroxyl groups or methoxyl groups at C-2, increased osteoblast ALP activity, but showed no any activity on osteoblast proliferation. The other compounds have no stimulatory effects on osteoblasts. It seems that the substituents at the C-1 and C-2 positions on the anthraquinone skeleton are related with their stimulatory effects on osteoblast bone formation. All seven of the anthraquinones have inhibitory effects on osteoclast TRAP activity and bone resorption. Their order of activity was **1**>**5**>**8**>**7**>**3**>**2**>**6**>**4**. The compounds **1** and **5**, which have stronger inhibitory activity on osteoclasts than the other compounds, have hydroxyl groups at the C-1 and C-8 positions. Taken together, although the structure–activity relationship was not conclusively demonstrated with these results, our present study suggested the importance of hydroxyl group in the C-1 and C-8 positions for the inhibitory activity of anthraquinone derivatives in osteoclastic bone resorption. 

Pathological research has indicated that osteoporosis is associated with many factors. Oxidative stress has an important impact on osteoclast differentiation and functions [13]. Some cytokines including macrophage colony-stimulating factor (M-CSF), IL-1, IL-4, IL-6, IL-11, and TNF-α produced by bone marrow stromal cells and osteoblasts also regulated osteoclast differentiation [14]. Anthraquinones such as physcion, rhein, emodin and diacerein showed significantly antioxidative activity, and pro-inflammatory cytokine antagonist activity [15,16,17,18]. Therefore, the inhibitory effects of anthraquinones on bone resorption may be associated with their antioxidative and anti-inflammatory activities.

## Conclusions

In the present study we have isolated seven anthraquinone derivatives and one coumarin from *M. officinalis* and evaluated their antiosteoporotic activity together with their structure–activity relationship using osteoblast and osteoclast cells. Among the seven anthraquinone derivatives isolated, compounds **4** and **5** showed significant stimulatory activity on osteoblast ALP activity in a dose-dependent manner, Compounds **1** and **5** showed stronger inhibitory effects on osteoclastic bone resorption. Our results also suggest the importance of hydroxyl groups at C-1 and C-8 in the anthraquinone derivatives for the inhibitory effects on bone resorption. Thus, it will be of interest to test further whether these anthraquinones exert antiosteoporotic effects *in vivo*, for example, in animal models of osteoporosis, to explore their therapeutic potential. This will provide further insight into the design of new approaches to osteoporosis.

## Experimental

### General

NMR spectra were obtained on a Bruker DRX-600 spectrometer operating at 600 MHz for ^1^H-NMR and 150 MHz for ^13^C-NMR. Peak positions are expressed in δ values referencec to TMS used as internal standard, and coupling constants *J* are in Hz. ESI-MS were recorded on a Varian MAT-212 mass spectrometer; column chromatography was performed on silica gel (200–300 mesh, Yantai, P. R. China) and Sephadex LH-20 (Pharmacia). The solvents used for isolation and purification were purchased from Sinopharm Chemical Reagent Co. Ltd. (Shanghai, P.R. China). α-MEM medium and fetal calf serum (FCS) were purchased from Gibco (U.S.A.). 1,25-Dihydroxyvitamin D_3_, dexamethasone, naphthol AS-BI phosphate, pararosaniline, Coomassie Brilliant Blue G-250 and 2-mercaptoethanol were purchased from Sigma (U.S.A.). The test compounds were dissolved in ethanol at a concentration of 10 mmol/L and diluted to an appropriate concentration using culture medium, and the final concentration of ethanol was adjusted to 0.01%. Ethylene glycol methyl ether, potassium sodium tartrate, disodium 4-nitrophenylphosphate, Triton^®^ X-100, and 4-nitrophenol were of domestic AR grade.

### Plant material

*M. officinalis* How (Rubiaceae, root, 2004021805) was obtained from Shanghai Hua Yu Chinese Herbs Co. Ltd. and identified by Prof. H.C. Zheng of the Department of Pharmacognosy, School of Pharmacy of the Second Military Medical University. The voucher specimens of these plants were deposited at the Herbarium of Department of Pharmacognosy, Second Military Medical University, Shanghai, P.R. China.

### Extraction and isolation

The dried roots (6 kg) of *M. officinalis* How were extracted with 75% ethanol three times under reflux, and the combined extracts were concentrated under reduced pressure. The residue obtained was suspended in water and successively partitioned with petroleum ether, ethyl acetate and butanol. The ethyl acetate extract (80 g) was subjected to silica gel column (10 cm i.d.×80 cm) chromatography eluted with a petroleum ether-ethyl acetate gradient (50:1→30:1→10:1→5:1→3:1→1:1) to give 6 fractions **Fr.1–Fr.6**. **Fr.** 2 (10.3 g) was subjected repeatedly to Sephadex LH-20 chromatography eluted with 100% methanol to yield compounds **1** (22 mg), **2** (180 mg), **3** (62 mg), and **4** (36 mg). Compound **5** (64 mg) was obtained by re-crystallization of the marc of **Fr.3** (103 mg) with ethyl acetate. **Fr.5** (1.3 g) was subjected repeatedly to Sephadex LH-20 chromatography eluted with 100% methanol to yield compounds **6** (45 mg), **7** (44 mg), and **8** (36 mg). 

*Physcion* (**1**). Yellow needles; ESI-MS: m/z 283 [M-H]^-^; ^1^H-NMR (CDCl_3_): δ 2.45 (3H, s, 6-CH_3_), 3.95 (3H, s, 3-OCH_3_), 6.70 (1H, d, *J* = 2.5 Hz, H-2), 7.09 (1H, d, *J* = 1.0Hz, H-7), 7.39 (1H, d, *J* = 2.6 Hz, H-4), 7.64 (1H, d, *J* = 1.0 Hz, H-5), 12.12 (1H, s, -OH), 12.31 (1H, s, -OH); ^13^C-NMR (CDCl_3_): δ 165.58 (C-1), 108.22 (C-2), 162.53 (C-3), 106.79 (C-4), 121.29 (C-5), 148.45 (C-6), 124.52 (C-7), 165.22 (C-8), 190.83 (C-9), 182.04 (C-10), 135.29 (C-4a), 110.29 (C-8a), 113.71 (C-9a), 133.25 (C-10a), 22.16 (-CH_3_), 56.08 (-OCH_3_). 

*Rubiadin-1-methyl ether* (**2**). Yellow needles; ESI-MS: m/z 267 [M-H]^-^; ^1^H-NMR (DMSO-D_6_): δ 2.16 (3H, s, 2-CH_3_), 3.79 (3H, s, 1-OCH_3_), 7.51 (1H, s, H-4), 7.82 and 7.91 (2H, m, H-6, H-7), 8.10 and 8.16 (2H, m, 5-H, H-8), 11.1 (1H, brs, 3-OH); ^13^C-NMR (DMSO-D_6_): δ 160.1 (C-1), 134.4 (C-2), 161.5 (C-3), 108.9 (C-4), 133.2 (C-4a), 126.0 (C-5), 133.7 (C-6), 134.5 (C-7), 126.6 (C-8), 135.0 (C-8a), 182.5 (C-9), 108.9 (C-9a), 180.1 (C-10), 133.0 (C-10a), 9.5 (CH_3_), 60.5 (OCH_3_). 

*2-Hydroxy-1-methoxyanthraquinone* (**3**). Orange-red needles; ESI-MS: m/z 253 [M-H]^-^; ^1^H-NMR (CDCl_3_): δ 4.0 (3H, s, 1-OCH_3_), 7.8 (2H, m, H-6, H-7), 8.1 (1H, s, *J* = 3.8 Hz, H-4), 8.3 (2H, m, H-5, H-8); ^13^C-NMR (CDCl_3_): δ 146.63 (C-1), 155.57 (C-2), 120.26 (C-3), 125.79 (C-4), 127.08 (C-5), 133.86 (C-6, C-7), 126.86 (C-8), 182.72 (C-9), 182.11 (C-10), 132.96 (C-11), 134.47 (C-12), 125.70 (C-13), 127.53 (C-14), 62.31 (-OCH_3_). 

*1,2-Dihydroxy-3-methylanthraquinone* (**4**). Yellow needles; ESI-MS: m/z 254 [M-H]^-^; ^1^H-NMR (DMSO-D_6_): δ 2.067 (3H, s, 3-CH_3_), 7.249 (1H, s, H-4), 7.9 (2H, m, H-6, H-7), 8.12 (1H, m, H-5), 8.19 (1H, m, H-5), 11.22 (1H, s, 2-OH); ^13^C-NMR (DMSO-D_6_): δ 162.80 (C-1), 162.42 (C-2), 133.00 (C-3), 107.32 (C-4), 126.36 (C-5), 134,54 (C-6), 134.44 (C-7), 126.69 (C-8), 186.26 (C-9), 181.82 (C-10), 131.74 (C-11), 132.89 (C-12), 117.32 (C-13), 108.98 (C-14), 8.04 (-CH_3_).

*1,3,8-Trihydroxy-2-methoxyanthraquinone* (**5**). Orange-red needles; ESI-MS: m/z 285 [M-H]^-^; ^1^H-NMR (DMSO-D_6_): δ 3.856 (3H, s, -OCH_3_), 7.268 (1H, s, H-4), 7.341 and 7.355 (1H, d, *J* = 8.4 Hz, H-7), 7.666 and 7.678 (1H, d, *J* = 7.2 Hz, H-5), 7.751,7.764 and 7.777 (1H, t, *J*_1_ = *J*_2_ = 7.8, H-6), 11.201 (br, 3-OH), 12.027 (8-OH), 12.180 (1-OH); ^13^C-NMR (DMSO-D_6_): δ 156.81 (C-1), 139.78 (C-2), 157.72 (C-3), 109.34 (C-4), 119.11 (C-5),136.96 (C-6), 124.37 (C-7), 161.21 (C-8), 190.70 (C-9), 180.84 (C-10), 133.20 (C-11), 115.82 (C-12), 110.11 (C-13), 129.11 (C-14), 60.16 (-OCH_3_). 

*2-Hydroxymethyl-3-hydroxyanthraquinone* (**6**). Yellow needles; ESI-MS: m/z 253 [M-H]^-^; ^1^H-NMR (DMSO-D_6_): δ 4.6 (2H, s, 15-CH_2_), 5.4 (1H, s, 16-OH), 7.5 (1H, s, H-1), 7.9 (2H, m, H-5, H-8), 8.2 (2H, m, H-6, H-7), 8.3 (1H, s, H-4), 11.1 (1H, t, J = 7.1Hz, 3-OH); ^13^C-NMR (DMSO-D_6_): δ 126.2 (C-1), 125.0 (C-2), 159.6 (C-3), 111.2 (C-4), 126.5 (C-5), 133.8 (C-6), 134.3 (C-7), 126.5 (C-8), 181.4 (C-9), 182.5 (C-10), 133.2 (C-11), 133.0 (C-12), 125.0 (C-13), 136.3 (C-14), 57.7 (-CH_2_OH). 

*2-Methoxyanthraquinone* (**7**). Yellow needles; ESI-MS: m/z 237 [M-H]^-^; ^1^H-NMR (DMSO-D_6_): δ 3.954 (3H, s, -OCH_3_), 7.778 (2H, m, H-6 or H-7), 8.282 (2H, m, H-5, H-8), 8.338 (1H, d, *J* = 8.0 Hz, H-4), 8.384 (1H, dd, *J* = 8.0, 1.7 Hz, H-3), 8.890 (1H, d, *J* = 1.7 Hz, H-1); ^13^C-NMR (DMSO-D_6_): δ 110.5 (C-1), 164.7 (C-2), 121.5 (C-3), 130.1 (C-4), 127.4 (C-5), 133.8 (C-6), 134.3 (C-7), 127.7 (C-8), 183.6 (C-9), 182.3 (C-10), 134.2 (C-11), 134.1 (C-12), 136.3 (C-13), 127.5 (C-14), 56.3 (-OCH_3_). 

*Scopoletin* (**8**). Yellow needles; ESI-MS: m/z 191 [M-H]^-^; ^1^H-NMR (DMSO-D_6_): δ 3.81 (3H, s, -OCH_3_), 6.20 and 6.22 (1H, d, *J* = 9 Hz, H-3), 6.78 (1H, s, H-8), 7.20 (1H, s, H-5), 7.88 and 7.90 (1H, d, *J* = 9 Hz, H-4); ^13^C-NMR (DMSO-D_6_): δ 160.6 (C-2), 111.6 (C-3), 144.3 (C-4), 109.5 (C-5), 145.1 (C-6), 151.0 (C-7), 102.7 (C-8), 149.4 (C-9), 110.5 (C-10), 55.9 (-OCH_3_). 

### Cell cultures

Wistar rats, which were 3-4 days old, were purchased from the Experimental Animal Center of the Second Military Medical University, Shanghai, P.R. China. Primary osteoblastic cells were prepared from the calvarias of newborn rats according to the literature [19]. These cells had typical properties of osteoblasts such as alkaline phosphates activity. Rat bone marrow cells were collected as described by Zambonin *et al.* [20]. Primary osteoblastic cells (1×10^8^/L) and bone marrow cells (1×10^9^/L) were co-cultured in α-MEM medium containing 10% FCS, 1,25-dihydroxyvitamin D_3_ (10 nmol/L) and dexamethasone (100 nmol/L) at 37°C in a humidified atmosphere of 5 % CO2 for 10 days in 6-well culture dishes (1.5 mL per well). Prior to plating the cells, a cover glass (5 × 5mm) or bone slices (40 µm thick) were placed into culture dishes. The formation of osteoclast-like MNCs (multinucleated osteoclasts) was confirmed by the staining of TRAP and resorption pit formed on bone slices.

### Assay for osteoblast proliferation and alkaline phosphatase (ALP) activity

The primary osteoblasts in neonatal rat calvarias cultures were treated with test substances or control (ethanol, final concentration 0.01% v/v) for 48 h, and prior to the end of culture, MTT was added to each well and incubated for 4 h after which the medium was discarded, and 150 µL of DMSO was added to each well. The cells were incubated for 20 min. The UV absorbance was measured at 540 nm at ELx 800 universal microplate reader (Bio-Tek) and used as an indicator of osteoblast proliferation. Primary osteoblasts were plated at 2×10^4^ cells/well in 96-well dishes, and treated with test substances or control for 6 days. The ALP activity was measured according to the literature [8]. Total protein was assayed by the method of Bradford [21]. The activity of alkaline phosphatase was expressed as micromoles of 4-nitrophenol liberated per milligram protein.

### Assay for osteoclastic TRAP activity

Primary osteoblast (1×10^5^ cells/mL) and marrow cells (1×10^6^ cells/mL) were placed on a 96-well plate, and cultured for 8 days, then treated with tested compounds for 48 h. After cell cultures, the cells were washed twice with PBS buffer and the TRAP activity of osteoclasts was measured as described previously [22]. The samples and standards were diluted in NaOH 20 mmol/L, and the absorbance was measured at 405 nm. The nanomolar number of 4-nitrophenol in each well was calculated and at the same time, the positive cells for TRAP were counted. The activity of TRAP was expressed as nanomoles of 4-nitrophenol liberated per minute per 100 osteoclasts.

### Determination of bone resorption pit

Primary osteoblast (1×10^5^ cells/mL) and marrow cells (1×10^6^ cells/mL) were placed on a 96-well plates containing bone slices in the bpresence of test compounds or control vehicle (ethanol, final concentration 0.01%), cultured for 12 days. Bone slices were ultrasonicated in NH_4_OH to remove adherent cells, stained with 0.1% toluidine blue solution. Resorption pits were observed and the pit area was quantified with image analysis software (Leica Q550IW, Germany) connected to a light microscope.
